# Patients’ Perspective of Medication Safety in a Structurally Burdened Healthcare System: A Netnography-Based Qualitative Analysis

**DOI:** 10.3390/healthcare14121784

**Published:** 2026-06-20

**Authors:** Barbara Báldy, Zoltán Cserháti, Judit Lám

**Affiliations:** 1 Interdisciplinary Social Sciences Doctoral Program, Mental Health Sciences Division, Doctoral College, Semmelweis University, 1085 Budapest, Hungary; 2 Health Services Management Training Centre, Semmelweis University, 1125 Budapest, Hungary; cserhati.zoltan@emk.semmelweis.hu (Z.C.); lam.judit@emk.semmelweis.hu (J.L.)

**Keywords:** medication safety, patient safety, patient perspective, netnography, grounded theory, healthcare access, communication

## Abstract

**Highlights:**

**What are the main findings?**

- Hungarian patients’ online narratives identify access barriers (long waits, brief consultations, rural service gaps) and communication failures (incomplete medication information, conflicting advice, disrespectful encounters) as the dominant threats to medication safety.
- Community pharmacists are largely absent from patients’ mental models of medication safety, despite being structurally well-positioned to address the very access and communication gaps patients report.

**What are the implications of the main findings?**

- Medication safety interventions in structurally burdened health systems should simultaneously target capability (patient awareness of pharmacy services), opportunity (access pathways), and motivation (trust-building communication) rather than focusing on information provision alone.
- Netnography combined with grounded theory-informed methodology is a transferable and productive approach for capturing authentic, unsolicited patient perspectives on medication safety in settings where formal institutional communication is perceived as inadequate.

**Abstract:**

**Background/Objectives**: Medication-related harm is a leading global patient safety challenge, yet patients’ lived experiences of medication safety remain underexplored in Central and Eastern European healthcare systems, where structural constraints significantly shape everyday medication use. **Methods**: This study provides an in-depth qualitative analysis of Hungarian patients’ online narratives, building on a prior netnographic mixed-methods study. Using grounded theory-informed principles and a patient-centred medication safety framework, we inductively analysed 5174 publicly accessible Hungarian-language comments posted on health forums and social media platforms between August 2020 and August 2023. The COM-B model was applied as a secondary lens to map findings onto modifiable behavioural determinants. **Results**: Access to services and communication emerged as the dominant medication safety concerns. Patients reported long waiting times, limited rural emergency services, and brief consultations leading to delayed or inadequate treatment. Communication gaps included insufficient information on medication duration, side effects, and follow-up, as well as conflicting advice from multiple sources, all of which eroded trust and prompted treatment discontinuation or reliance on informal online communities. Community pharmacists were largely absent from patients’ mental models of care, representing a significant missed opportunity given their accessibility. Less frequently mentioned were medication shortages, healthcare professional workload, and systemic safety culture. **Conclusions**: Clear, respectful communication and timely access to care are central to medication safety from the patient perspective. Netnography combined with a grounded theory-informed methodology offers a valuable approach for capturing authentic patient perspectives in structurally burdened healthcare systems, with findings relevant beyond the Hungarian context.

## 1. Introduction

Medication-related harm is a leading cause of preventable injury in healthcare systems worldwide and has been recognized by the World Health Organization (WHO) as a key patient safety priority under its “Medication Without Harm” initiative [1]. Within this context, medication safety has emerged as a focal area not only for clinicians and health systems, but increasingly for patients themselves [2,3]. While medication safety initiatives have traditionally emphasized institutional processes—such as prescribing accuracy, pharmacovigilance, and system-level error reduction—there is growing recognition that patients play a vital role in ensuring safe medication use [4,5,6,7]. Their experiences, concerns, and coping strategies form a critical yet underexplored dimension of the medication safety landscape [5].

Despite this paradigm shift, there remains a limited understanding of how patients define and experience medication-related safety risks in their daily lives, particularly outside formal care settings [5]. This gap is especially evident in Central and Eastern European countries such as Hungary, where patient safety research remains sparse and medication safety has rarely been examined from the patient perspective. Community pharmacy, as the most accessible tier of the health system, represents a particularly underexamined safety interface in this region, despite its potential to mitigate the very access and communication gaps patients report.

Within this landscape, the role of community pharmacists as frontline medication safety advocates has gained increasing attention in social and administrative pharmacy research. Pharmacists interact with patients at a critical juncture—the point of medication dispensing—where counselling, adherence support, and safety checks can meaningfully reduce harm [8]. Yet in many Central and Eastern European health systems, pharmacists’ advisory roles remain underrecognized by patients, and their potential contribution to medication safety is constrained by systemic, regulatory, and cultural factors. Understanding how patients perceive and navigate medication safety—including their awareness (or lack thereof) of pharmacist services—is essential for designing effective, patient-centred interventions.

Netnography enables researchers to study how individuals discuss sensitive topics like medication concerns, therapeutic doubts, or physician encounters in peer-driven spaces that are often less accessible through traditional surveys or interviews. This methodology is especially useful in structurally burdened health systems, where patients may seek support or validation in digital communities rather than through official channels [9]. In our prior work using a netnographic dataset, we found that Hungarian online conversations about medications were overwhelmingly driven by patients, with only minor input from relatives and healthcare professionals [5].

Using the patient-centered medication safety framework developed by Giles et al. [2], we previously identified two dominant themes in the Hungarian data: *Access to services* and *Communication*. These categories accounted for the majority of patient concerns, particularly those involving prescription medications across gynecology, internal medicine, and gastroenterology [5]. Notably, medications classified under ATC codes G (genito-urinary and hormonal preparations) and A (alimentary tract and metabolism) were most frequently mentioned, mirroring the clinical domains patients engaged with online [5]. Posts reflected intense emotional responses to systemic access barriers—such as difficulty obtaining prescriptions or lack of follow-up—and to communication gaps, including unclear instructions, insufficient counseling, or feeling dismissed by providers [5]. These findings support growing international evidence that medication safety is shaped not only by clinical accuracy, but also by patients’ ability to access care and feel heard in their treatment journeys [10,11].

While the applied analytical framework functioned well in the Hungarian context, the imbalance between certain categories suggests that local specificities may modify the applicability of international models [5]. Similar imbalances between analytical categories have also been observed in other health systems, suggesting that patient perceptions and systemic constraints can reshape the applicability of international models [4,12]. These results are relevant not only for the Hungarian context but also internationally, particularly for populations where the availability of publicly funded care is limited or where structural weaknesses affect the healthcare system. The Hungarian case offers a unique intersection of systemic challenges and patient-level coping strategies, making it potentially instructive for other countries facing similar difficulties.

The present article builds on our previous mixed-methods analysis [5] by offering an in-depth qualitative interpretation of the same dataset. Using grounded theory-informed methodology as our analytic approach, we aim to inductively explore patients’ digital narratives without imposing predefined categories, focusing instead on how medication safety concerns are constructed, expressed, and negotiated in online settings. This methodological lens is particularly appropriate for exploring social phenomena that emerge where formal institutional communication is inadequate, and where digital communities become alternative spaces for advice, support, and error mitigation.

### Research Questions

This article addresses the following qualitative research questions:

1. Is netnography an appropriate methodology for exploring patient perspectives related to medication safety within health research?
2. What are the principal medication safety issues highlighted by patients in online communication?

The full list of the study’s broader research questions is provided in our previous publication [5].

Beyond the Hungarian context, this study aims to generate exploratory, theory-informed insights into how medication safety is experienced by patients in structurally constrained healthcare systems, where access barriers and communication gaps shape everyday medication use.

## 2. Materials and Methods

### Study Setting: The Hungarian Healthcare System

Hungary operates a compulsory social health insurance system, primarily financed through payroll contributions and managed by the National Health Insurance Fund (NEAK, Budapest, Hungary). All residents with insurance are entitled to primary care through assigned general practitioners (GPs), specialist outpatient care via referral, and inpatient hospital services. However, structural underfunding, workforce shortages—particularly in rural areas—and long waiting times for specialist consultations have prompted a growing segment of the population to seek care in the private sector, which operates on an out-of-pocket or voluntary insurance basis [13].

General practitioners serve as gatekeepers to the specialist care system. While GP visits are generally accessible, the brevity of consultations (often limited to 5–10 min due to high patient volumes) and the difficulty of reaching GPs outside of scheduled office hours are well-documented challenges [14]. Community pharmacies are widely accessible across the country and serve as a primary point of contact for medication dispensing. Recent regulatory developments—including the introduction of pharmaceutical care guidelines (gondozási irányelv)—have begun to expand pharmacists’ roles beyond dispensing toward chronic disease management and patient education, though uptake remains uneven.

This study represents the qualitative follow-up to a mixed-method netnographic analysis of Hungarian patients’ online discussions about medication use. In the previous part of the study, we described the design, sampling, and application of a patient-centered medication safety framework (developed by Giles et al. [2]) (Table 1) to categorize and quantify safety-related comments in Hungarian-language health forums and social media [5]. The current section builds on that foundation through an in-depth inductive analysis of patients’ narratives using grounded theory-informed principles.

We employed a grounded theory-informed qualitative approach to identify patterns in patients’ expressions, coping strategies, and perceptions related to the previously identified two main medication safety domains: Access to services and Communication. While we did not add new top-level codes beyond those defined in the original medication safety framework, the analytic approach was inductive, iterative, and theory-generating. Rather than assigning predefined subcategories, we closely examined the textual content of patient comments within each domain, allowing recurring themes and conceptual categories to emerge organically from the data. This strategy aligns with grounded theory’s emphasis on constructing explanatory models from lived experience and is well suited for analyzing unstructured patient narratives in online environments [15].

While the Giles et al. (2020) [2] framework provided the initial structural scaffold, the analytical process was deeply rooted in grounded theory-informed principles. This approach ensured that the analysis remained open to discovery, moving beyond simple deductive categorization. Using the constant comparative method, we iteratively cross-referenced new data with existing codes, allowing for a ‘bottom-up’ emergence of sub-themes that reflect the unique socio-cultural realities of the Hungarian healthcare context.

We adopted a constructivist, interpretivist epistemological position, treating patients’ online narratives as situated accounts of lived experience rather than objective records of events. Our analytic logic was abductive: the patient-centred framework of Giles et al. [2] provided an orienting structure and ensured comparability with our companion mixed-methods study [5], while sub-themes were developed inductively from the data. We acknowledge that this is not classical (Glaserian) grounded theory, which seeks theory emergence with minimal reliance on pre-existing frameworks; rather, we drew selectively on grounded-theory analytic techniques (constant comparison, theoretical sensitivity, and analytic memoing) to generate sub-themes within the framework’s top-level domains. In this respect the approach also shares features with the framework method and with hybrid deductive–inductive thematic analysis. We therefore use “grounded-theory-informed” to denote this use of grounded-theory techniques rather than a claim to the full methodology.

The dataset consisted of 5174 comments posted between August 2020 and August 2023. Data were retrieved from the publicly accessible Hungarian-language online environment using the SentiOne social-listening platform (Automate version 224, Poland), selected for its comprehensive coverage of Hungarian public online content. Sources comprised open health forums, other public websites, and public Facebook pages, while closed or private Facebook groups and non-public platforms were deliberately excluded, as they do not constitute publicly available data. This sampling frame necessarily favours users who participate in open, searchable forums and may underrepresent those active only in closed communities—a selection bias we return to in the Limitations. The platform, the keyword strategy (including 93 exclusion terms), and the stepwise reduction from 122,772 retrieved to 5174 relevant comments are reported in full in our companion mixed-methods paper [5]. Only comments related to oral medications were included, as these are commonly used in outpatient care and require high levels of patient involvement [5].

Because the analysis drew on a fixed, retrospectively archived dataset rather than iteratively sampled data, theoretical sampling in the classical grounded-theory sense was not applicable. Thematic saturation was therefore assessed analytically: applying the constant comparative method, coding continued until no new sub-themes emerged within the Access to services and Communication domains, at which point the conceptual categories were judged stable and saturated.

To support the transparency and rigour of the inductive analysis, coding was undertaken by the first author and reviewed in full by the third author, with interpretations compared through constant comparison. Rather than relying on a numerical inter-rater coefficient—of limited applicability to interpretive, theory-generating coding, where the aim is the shared construction of meaning rather than convergence on predefined categories—analytic consistency was established through investigator triangulation, with discrepancies discussed until consensus was reached. Throughout the analysis, the researchers maintained analytic and reflexive memos documenting emerging sub-themes and coding decisions, which informed the constant comparative process.

**Figure 1:**
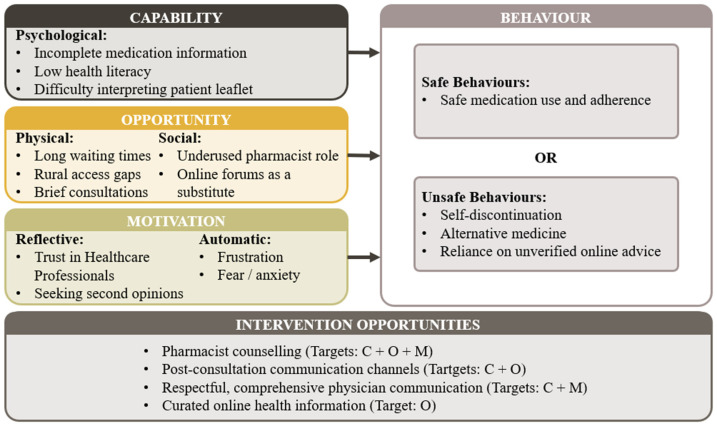
COM-B mapping of Medication Safety Experiences of the Hungarian Patients. The mapping is a post hoc interpretive synthesis of the inductively derived sub-themes.

To move beyond descriptive analysis, the COM-B model [16,17] was integrated as a secondary analytical lens for behavioural diagnosis. The mapping followed a two-stage analytical pathway. Sub-themes were first developed inductively within the Giles et al. [2] domains, after which the COM-B model was applied as a secondary, post hoc deductive lens. Each sub-theme was examined and assigned, through discussion and consensus, to the COM-B component it most directly reflected. For example, gaps in medication information were mapped to psychological capability, systemic access barriers to physical opportunity, the under-recognised role of pharmacists to social opportunity, and trust-related experiences to reflective motivation. The mapping was therefore interpretive and post hoc rather than inductively emergent, serving to organise the inductively derived sub-themes into behaviourally meaningful determinants (Figure 1 presents the resulting synthesis).

## 3. Results

The following section presents the key themes identified through netnographic analysis of online patient discussions, organised around the most prominent determinants of medication safety: access to services and communication, followed by less frequently mentioned framework elements.

### 3.1. Access to Services

Patients in online discussions often report difficulties in accessing necessary healthcare services and obtaining their prescribed medication in a timely manner. One of the most frequently mentioned problems is long waiting times—often lasting weeks or even months—which can lead to the progression of their medical conditions. Delays in care are also common when patients are unable to access emergency services during weekends or public holidays, such as in cases of emergency contraception. These challenges are particularly prevalent in rural areas, where access to public healthcare and emergency care is limited, leaving patients more vulnerable. In such situations, patients tend to be proactive, attempting to resolve their medication needs on their own; however, as time passes without success, they often become increasingly frustrated and stressed. Even when they are able to access services, many report that physicians have only “five minutes” for a consultation, which raises doubts about whether the prescribed treatment is appropriate—especially for those with a lengthy medical history or complex health problems.

*“I had hellish migraines every month for years when I had my period. They went away when I stopped taking birth control and was prescribed a different blood pressure medication. Ten years later, they realized I shouldn’t have been taking them together.”*

A common problem arises when patients are unable to access healthcare services and therefore cannot obtain essential information about their medication. In many cases, they did not—or could not—ask their questions during in-person consultations with their physician. Others, particularly those with long-term health conditions, are unsure which service they need to approach. In such situations, they often turn to alternative sources of information. Many seek advice in patient–physician forums, where they describe their problems and expect direct recommendations on which medication to use. These expectations are often unrealistic and can lead to disappointment, as such forums are not intended to address complex medical issues. It is also common for patients to have insufficient information about their current medication—sometimes because they do not know how, or are unable, to contact their physician. As a result, they search for the missing information online or ask other patients. In many instances, they are unclear about why they are taking a particular medication or how long they should continue it. This suggests that they primarily expect such information from physicians, rather than from pharmacists.

*“Dear Doctor, My question is: how long can Normix tablets be taken? (intestinal flora imbalance, minor inflammation in the colon described as atypical inflammation) (complete upper and lower endoscopy, capsule endoscopy also performed, negative calprotectin) I had to take Azi Sandoz for an upper respiratory tract infection, I have just finished the course a month ago and would now like to start taking Normix again. Can I take for one week, two pills twice a day every month for months on end? Thank you very much!”*

When patients have negative interactions with the healthcare system—such as feeling disrespected by physicians, perceiving that their concerns are dismissed, or not receiving answers to their questions—they tend to adopt one of two problem-solving strategies: either they stop trying to access the services altogether, or they seek alternative routes, such as the private sector, other institutions, their general practitioner or, in some cases, even foreign healthcare providers. In such circumstances, patients may be unable to obtain the appropriate medication or experience significant delays in doing so. The high cost of private healthcare excludes many patients; however, some recognize that, in the long run, investing more money can be worthwhile if it allows timely access to appropriate medication and prevents the progression of their condition—which could also become costly. It is also common for patients to seek a second opinion, which often yields positive results and reinforces their sense of control and involvement in their own care. It is also common for patients to try to reach services by phone or e-mail, but in many cases these telemedicine routes are unsuccessful or difficult to access.

*“These private clinics are very good solutions [in the need of emergency contraception pill]. Unfortunately, [due to an incident] I was quickly forced to use them, but I can highly recommend them if you don’t mind spending 10,000 forints just for a prescription and are pressed for time. You fill out the form, they call you, ask you a few questions, and that’s it. The doctor was incredibly kind and urged me to go and get the prescription immediately and take it on the spot, and to call him if anything happened. I don’t think I would have been so lucky at a public clinic.”*

Another issue arises when patients report that physicians prescribe medication without conducting the necessary tests or examinations, leading them to feel that proper healthcare services are lacking. The most frequently mentioned omissions were laboratory tests and imaging procedures. These concerns were raised most often in relation to the prescription of contraceptive pills. In many cases, patients only become aware of these shortcomings later—sometimes when they experience subsequent health problems that they believe are connected to medication.

*“‘You don’t need a damn blood test before they prescribe you birth control pills. They might prescribe a hormone to someone with diabetes, thyroid disease, or an autoimmune disorder, which could even make them infertile. But one thing is certain: it won’t make you lose weight, and your acne won’t go away.’ That’s how the doctor happily prescribed the pills to me back then. It was only nearly 20 years later that my illness was discovered. I shouldn’t have been taking them.”*

In some cases, when patients are unable to access healthcare services, they turn to alternative medicine, such as herbal remedies, which can pose health risks. This also occurs when patients attempt to self-diagnose and treat themselves with alternative products. In general, within online discussions, other patients tend not to validate these types of actions and instead encourage their peers to seek professional medical assistance.

*“I used artificial intelligence to find an herbal equivalent to Frontin because I need it but didn’t want to become dependent on it. It works, and I’m satisfied with my herbal tablets, which are available at the pharmacy.”*

### 3.2. Communication

Regarding medication safety, a common issue reported by patients is that physicians often fail to provide essential information during consultations. This includes not specifying how long the medication should be taken, not mentioning possible side effects, not explaining the expected outcomes, and not advising on what actions the patient should take. As a result, patients may later face problems that could have been prevented, which can undermine their trust in healthcare providers. Another frequent concern is when physicians do not discuss how long it may take to see results or when patients should return for a follow-up appointment regarding their medication. Such incomplete communication can cause significant confusion: patients may be unsure about the next steps, how long it is normal for symptoms to persist, or when to seek further medical advice if there is no improvement. Overall, these miscommunications can lead patients to feel insecure about their treatment, fearful of making mistakes or harming themselves, and ultimately more vulnerable.

*“Dear Doctor, I am 51 years old and my doctor has recommended Ryeqo medication because since last November I have been experiencing prolonged bleeding and intermenstrual bleeding due to an intramural myoma. After five months of Norithesteron treatment, my symptoms have returned. […] I have read that Ryeqo is also not recommended for women over 50. I would like to ask for your professional opinion. Could Premens tablets reduce the number of days of bleeding? Can I take them at the same time as Norihesteron? […] Thank you for your reply.”*

A recurring problem is that patients are only able to communicate with physicians during in-person consultations, leaving no opportunity to ask follow-up questions or address uncertainties afterwards. This becomes particularly problematic when patients later encounter conflicting information—whether from a second opinion, online sources, or the patient information leaflet—and are unsure how to proceed. Such situations are common with contraceptive pills, where a physician may state that they are safe to use in certain medical histories, yet the patient information leaflet suggests otherwise. These discrepancies can undermine trust in medical science, medication safety, and healthcare services, leading patients to worry about their health.

In response, some patients stop taking their medication without consulting a physician, seek a second opinion from another healthcare provider, or search for further information online. Once trust is eroded, it can be difficult to restore; for example, when patients believe that two medications have a dangerous interaction—such as antibiotics and contraceptive pills—even a second opinion may not reassure them. Although uncommon, some patients go beyond reading the information leaflet and attempt to verify physicians’ advice by consulting scientific papers. While misinterpretation of such sources can be misleading, this practice can also improve health literacy, provide emotional reassurance, and help patients feel more in control of their care.

*“I told my gynecologist that I had a cerebral thrombosis in the past, and he prescribed Lorell tablets for me, but the patient information leaflet specifically states that this medication is not recommended in such cases! What do you think? Am I worrying unnecessarily, or is there another safe method of contraception?”*

Another concern arises when physician–patient communication lacks respect. Many patients reported feeling that the physician did not take them seriously, displayed a lack of empathy, or communicated in an impatient or abrupt manner. In some cases, when patients asked questions about their medication, physicians were perceived as downplaying the issue—for example, minimizing the significance of side effects that were a source of worry and had a tangible impact on patients’ quality of life. Such communication problems can once again undermine trust and may lead to non-adherence or medication errors.

Conversely, when patients encounter physicians who explain their condition and treatment thoroughly, they are perceived as helpful, empathetic, and patient in their communication; the experience is comforting and fosters trust. This trust, in turn, increases the likelihood of correct medication use. In these situations, patients report feeling more in control and confident in the success of their treatment.

*“The most humiliating experience of my life was related to a female gynecologist, whom I visited after months of pain and gynecological complaints. Finally, I found a conscientious male gynecologist who took me seriously, was empathetic, and examined me thoroughly. By the time I got to him, my results were very bad. […] He really treated me like a loving, concerned grandfather and was genuinely happy for me when the worst-case scenario did not come true.”*

### 3.3. Less Frequently Mentioned Framework Elements

While access to services and communication emerged as the most prominent themes, patients also referred to a number of additional framework elements, although these were mentioned less frequently.

*Supplies of medication and appliances* were occasionally mentioned, most often in relation to shortages. Patients described situations where their prescribed medication was unavailable, and no generic equivalent was on the market. In such cases, they often had to call or visit multiple pharmacies, which caused considerable stress due to the uncertainty. In some instances, the substitute medication was more expensive, creating additional financial burdens.

*Healthcare professional factors* were also less frequently discussed, but some patients recognized that the healthcare system faces human resource shortages, which can affect physicians’ communication and perceived effort in relation to medication. Physicians were described as at risk of burnout and overload. In gynaecology, patients noted that the physician’s sex and age could influence their level of trust, often based on previous personal experiences.

*Role and responsibilities* were most often linked to physicians, particularly regarding decisions about contraindications and the choice of effective medication. In relation to pharmacists, two contrasting attitudes emerged: some patients did not see them as having a role in medication safety—possibly due to negative experiences or lack of awareness that pharmacists can be consulted about medication issues—while others expressed trust in them because of their professional education. Although patients are trying to be active in learning about their medication, they did not appear to recognize their own role in medication safety. They also did not discuss the relationship between physicians and pharmacists.

Finally, some framework elements were rarely associated with medication safety by patients. These included: *Medication safety culture, Medication policies and processes, Workload of health care professionals, Continuity of care, Computer systems and programs, Patient- and carer/relative-related factors* and *Dignity and respect* (as an explicit act).

## 4. Discussion

This study provides an in-depth qualitative interpretation of patients’ medication safety experiences in Hungary, highlighting access to services and communication as the central determinants shaping safe medication use. To deepen the theoretical interpretation of our findings, we applied the COM-B model of behaviour change^16^ as a secondary analytical lens. The COM-B framework posits that behaviour (B) is the result of interactions between capability (C), opportunity (O), and motivation (M). This model is widely used in pharmacy practice research and health behaviour interventions, and allows us to map patients’ medication safety experiences onto modifiable determinants of safe medication use. In response to our research question regarding the principal medication safety issues highlighted by patients, our findings suggest that access to services and communication are the most critical concerns, reflecting systemic barriers that undermine patients’ ability to use their medication safely and confidently.

Our findings suggest that patients’ medication safety behaviours are shaped by deficits across all three COM-B domains. Psychological capability was undermined by insufficient information provision during consultations—patients frequently lacked knowledge about medication duration, side effects, and expected outcomes, as well as the availability of counselling at the pharmacy (see Table 2, Communication sub-themes). Physical opportunity was constrained by systemic access barriers, including long waiting times, limited emergency services in rural areas, and brief consultations (see Table 2, Access sub-themes). Social opportunity was affected by the underrecognized role of community pharmacists and the reliance on informal online forums as alternative information sources. Reflective motivation was closely tied to trust: when communication was respectful and comprehensive, patients reported greater confidence and adherence, whereas dismissive encounters eroded motivation and led to treatment discontinuation or alternative care-seeking. Automatic motivation was implicated in emotionally driven responses such as impulsive medication cessation or turning to alternative medicine when frustration peaked. This mapping (Figure 1) suggests that medication safety interventions in Hungary—and comparable health systems—should address multiple COM-B components simultaneously rather than focusing solely on information provision.

Although this study is conducted in the Hungarian healthcare context, the key issues identified—access to healthcare, communication, and trust—are not unique to this setting. Similar challenges have been reported in other healthcare systems, suggesting that the findings have broader relevance [3,10,12,18,19]. The combined use of netnography and grounded theory-informed metodology proved particularly valuable, as it enabled the exploration of authentic patient voices in an online environment and allowed for the inductive development of insights that extend beyond the predefined Giles et al. [2] framework. This methodological approach highlights the importance of incorporating patients’ lived experiences into medication safety research. The application of grounded theory-informed principles was instrumental in identifying the ‘imbalance’ between the theoretical Giles categories and actual patient priorities in Hungary. By allowing the data to ‘speak’ without the constraints of a rigid deductive system, we were able to demonstrate that medication safety in this region is perceived primarily through the lenses of access and interpersonal trust, rather than institutional safety culture or technological failure. In response to our research question regarding the appropriateness of netnography as a methodology, our findings suggest that it offers a uniquely valuable lens for capturing authentic, unsolicited patient experiences of medication safety, particularly in contexts where formal institutional communication is perceived as inaccessible or inadequate.

Addressing the first research question more critically, netnography is particularly well suited to medication safety research because it captures unsolicited, naturally occurring accounts: patients discuss their concerns spontaneously and anonymously, free of the reactivity, social-desirability effects, and recruitment barriers that can constrain interviews, focus groups, or surveys—an advantage that is especially valuable for sensitive topics such as contraception and for patients who distrust formal institutions. At the same time, the method permits no probing, clarification, or member checking, meaning must be inferred from text alone, and identities and reported events cannot be verified (see Limitations and Table 3). These features make netnography especially effective for surfacing the lived, affective, and relational dimensions of medication safety that formal instruments often miss.

Because patients tend to focus on their illness during brief consultations and struggle to reach physicians afterwards, information gaps often surface only later. Targeted educational campaigns have sought to address these gaps, including WHO’s Medication Without Harm campaign [1], the Medication Safety Adventure Trail [20], and the Hungarian Necessary, Effective, and Safe Patient Cooperation in Pharmacies Programme [21].

When patients cannot access physicians, they often turn to online spaces, such as patient–physician forums. Their trust in the healthcare system stands on shaky ground: on the one hand, they are willing to ask physicians and show trust in medical science; on the other hand, this trust is easily eroded when communication in the healthcare system is inadequate. A recurring realization in discussions is that the problem lies less with individual physicians than with the healthcare system as a whole. Patients clearly express their desire to take action, to better understand their medication, and to be treated as equal partners. In forums, they encourage one another with a “keep going until you get an answer” attitude—an approach that should be equally encouraged and supported by the healthcare system itself.

The dominance of medications under ATC codes G and A in the narratives may be explained by the intimate nature of these conditions and their chronic progression. Patients often seek the anonymity of digital communities to discuss sensitive issues, such as emergency contraception or long-term gastrointestinal disorders, where formal institutional communication is perceived as inadequate or judgmental. This digital ‘safe haven’ allows for the expression of concerns that remain suppressed in clinical encounters.

Patients reported fewer issues with the daily medication routines once a prescription was given, as Hungarian pharmacists provide clear written instructions on medication packages.

From a social and administrative pharmacy perspective, our findings highlight a critical gap in patients’ awareness of pharmacists’ role in medication safety. While pharmacists were occasionally mentioned in positive terms—particularly regarding the clear written instructions provided on medication packages—many patients did not perceive pharmacists as accessible sources of medication counselling. This finding aligns with international literature showing that patients in transitional health systems often underutilize community pharmacy services for medication-related concerns [8]. The limited recognition of pharmacists in patients’ narratives highlights a missed opportunity within the medication safety system. Given their accessibility and expertise, community pharmacists could function as critical safety nodes, particularly in contexts where access to physicians is constrained.

The recent introduction of pharmaceutical care directives in Hungary (known as the “gondozási irányelv”) has created a policy framework for expanding pharmacists’ roles in chronic disease management, medication review, and patient education. The SZEBB Programme [21] (Necessary, Effective, and Safe Patient Cooperation in Pharmacies), developed by the Hungarian Chamber of Pharmacists, represents a targeted effort to bridge this gap. However, our data suggest that patient awareness of these expanded services remains limited.

The landscape of medication safety in Hungary is currently undergoing a transformative shift. Following the framework legislation of 2025, a new governmental decree issued in March 2026 (9/2026. BM decree) has formally introduced pharmacist-led prescribing for chronic medications. This allows pharmacists to renew prescriptions for patients. This policy aims to alleviate the burden on primary care and reduce the ‘access barriers’ identified in our study [22].

The policy considerations that follow extend beyond our empirical findings and should be read as interpretive recommendations rather than direct results of the analysis. Beyond efficiency, this reform may also contribute to the professional prestige and social recognition of pharmacists. International evidence suggests that transitioning from a dispensing role to a clinical provider fosters deeper patient trust. By positioning pharmacists as autonomous partners in chronic care, this change could help reposition the pharmacy from a point of sale toward a safety net within primary care. Because our dataset (2020–2023) predates these reforms, our findings describe the pre-reform baseline: they cannot speak to the reforms’ effects, but they document the access and communication barriers the reforms are intended to address and show that, as the roll-out begins, many patients remain unaware of these new competencies and still turn to informal online forums for access-related problems.

While the SZEBB programme [21] (Necessary, Effective, and Safe Patient Cooperation in Pharmacies) aims to enhance the professional visibility of pharmacists, our findings point to limited patient recognition of pharmacists as a source of medication counselling, a pattern we describe as “pharmacist-blindness.” This interpretation is consistent with the infrequent, largely physician-centred discussion of professional roles in our data and with the limited presence of healthcare professionals in the online discussions overall [5]. This gap in social opportunity suggests that simply expanding clinical competencies (e.g., via the 9/2026 BM decree) would likely need to be accompanied by targeted public awareness campaigns to reposition pharmacists as trusted medication safety advocates.

Strengthening the pharmacist’s position within the medication safety ecosystem would require coordinated action across the COM-B domains: enhancing patients’ awareness of pharmacist services (Capability), ensuring adequate staffing and consultation spaces in pharmacies (Opportunity), and building trust through positive interpersonal encounters (Motivation). Community pharmacists—given their accessibility, frequency of patient contact, and pharmaceutical expertise—are well positioned to serve as a safety net within structurally burdened primary care systems [8,23].

Although the COM-B mapping was applied interpretively and post hoc, it can be operationalised into concrete, multi-level, and testable intervention components. At the level of capability, patient-facing awareness initiatives (building on existing efforts such as the SZEBB programme [21]) could explicitly reposition the pharmacy as a site for medication counselling rather than dispensing alone, for example through standardised signposting at the point of dispensing, visible in-pharmacy information on available consultation services, and the inclusion of pharmacist contact details on prescription materials, with patient-reported awareness of pharmacy counselling services as a measurable outcome. At the level of opportunity, establishing semi-private consultation spaces and protected time for structured medication reviews [23] would create the conditions for meaningful engagement, while pharmacist-led digital channels (e.g., secure messaging or telepharmacy consultations) could meet patients in the same online spaces to which they currently turn for unmet information needs; uptake of these services and the proportion of medication queries resolved at the pharmacy rather than online would provide testable indicators of effect. At the level of motivation, proactive counselling offered at each medication change (the precise junctures at which patients in our data reported missing information) together with continuity through a named pharmacist for chronic patients under the newly introduced prescribing competencies, could foster the interpersonal trust that underpins safe medication use, with patient-reported trust and self-reported adherence as candidate outcomes. Framed in this way, the mapping yields discrete, evaluable hypotheses that could be tested in future implementation studies.

Another challenge concerns medication shortages, which patients frequently report. In such cases, they find themselves in situations of uncertainty, fear, and frustration. Better information and guidance are urgently needed when shortages occur, as these situations reveal possible structural flaws in the medication supply system.

Certain patient groups are at higher risk of medication errors—notably those with minority ethnic backgrounds, low socio-economic status and low health literacy [11,24]. Raising awareness of these disparities among healthcare staff is crucial for developing targeted interventions to reduce inequalities [11,24]. At the same time, uniform protocols are still lacking in some areas. In this study, problems emerged particularly in the field of contraception, where differing medical opinions and conflicting information caused mistrust and anxiety among patients.

It is important to note that patients consistently respond positively to good communication. They are grateful for clear explanations, empathy, and support, which in turn foster compliance and adherence. Even after negative experiences, many remain confident that they may still encounter respectful and effective communication with another healthcare provider. Ultimately, trust, built on effective communication, appears closely linked to adherence and safe medication use. Building on this, our findings highlight that improving medication safety requires not only better information provision, but also a broader, system-level approach. From an intervention perspective, medication safety strategies should therefore address structural opportunity (e.g., access pathways), social opportunity (e.g., pharmacist engagement), and motivation (e.g., trust-building communication) in an integrated manner. Addressing medication safety from a patient-centred perspective therefore requires not only improving clinical accuracy, but also redesigning access pathways and communication practices to better align with patients’ lived experiences in real-world settings.

### Limitations

This study has several limitations. First, our methodology relied on publicly accessible digital platforms, which inherently introduces selection bias. Netnographic data collection assumes a certain level of digital literacy and internet access, potentially underrepresenting older populations or marginalized socioeconomic groups who may experience the most severe barriers to care and medication safety. Because public online comments carry no verified demographics, we cannot confirm whether groups at highest risk of medication-related harm—older adults, men, and lower-SES users—are adequately represented, as these groups are also less active in open online discussions, their perspectives may be underrepresented. Future studies using interviews, surveys, or pharmacy-based designs could establish how far these findings extend to them. Furthermore, participants in online forums are often motivated to post by negative experiences or acute problems, which may overrepresent system failures compared to successful therapeutic encounters. Beyond individual motivations, online health communities tend to normalise and amplify the disclosure of negative experiences; future studies could address this through triangulation with interviews or surveys to capture a more balanced range of experiences.

Second, the analysis is strictly limited to the patients’ subjective perspectives. Without access to clinical records, it was not possible to medically validate whether the reported incidents were actual prescribing errors, miscommunications, or misinterpretations of medical advice by the patients. Additionally, demographic information (such as age, gender, exact location, or socioeconomic status) was largely unavailable, preventing targeted subgroup analyses, although we assume that topics like contraception were predominantly discussed by women. We did not undertake a dedicated pharmacy-specific keyword search or stratified sub-analysis to quantify pharmacist visibility, the account of “pharmacist-blindness” therefore rests on the qualitative pattern of role-related comments rather than a targeted frequency analysis, and a focused sub-analysis of pharmacist mentions represents a valuable direction for future work.

Third, the data collection period spanning from August 2020 to August 2023 overlaps significantly with the COVID-19 pandemic. The systemic disruptions caused by the pandemic—such as restricted in-person care, the abrupt shift to telemedicine, and prolonged waiting lists—likely amplified the access barriers reported by patients.

Finally, our research was restricted to oral medications. While these are the most common in outpatient care, this focus excludes complex administrative routes (e.g., injectables, inhalers, biologics), which often carry different and potentially higher risks for patient-led administration errors.

## 5. Conclusions

Hungarian patients’ digital narratives reveal that medication safety is experienced primarily through the lenses of access and interpersonal trust rather than institutional safety culture or technology. Community pharmacists, though structurally well-positioned, remain an underused safety resource in patients’ mental models of care. Strengthening pharmacist visibility through coordinated capability, opportunity, and motivation interventions (supported by recent Hungarian regulatory changes) offers a concrete path toward patient-centred medication safety improvement. Netnography combined with grounded theory-informed methodology proved a productive methodological pairing for surfacing these patterns and is transferable to other structurally burdened health systems.

## Figures and Tables

**Table 1:** The original framework based on Giles et al. [2].

Issue	Definition
1. Access to services	Access to services that provide prescriptions and medicines, and/or access to health care professionals who can give you information about medicines.
2. Communication	Effectiveness of the exchange and sharing of information about medicines between hospital and general practice, staff, patients, groups, departments, and services.
a. Communication between HCPs	Lack of effective communication in supplies of medication, changes in dose, formulation.
b. Communication between healthcare professionals and patients	Lack of appropriate information about medication use, such as medication changes, length of treatment, lack of listening to patient’s concerns about their medication (including medication errors).
3. Computer systems and programs	Failures of systems, poor design, and lack of interfacing between systems.
4. Continuity of care	Continuity of healthcare professionals who deal with medicines (e.g., locum pharmacists and GPs).
5. Dignity and respect	Associated with feeling comfortable, in control and valued.
6. Healthcare professional factors (This tended to focus on the knowledge of HCPs from the patient’s point of view, but also about attitudes.)	Characteristics and knowledge of the person delivering care that may contribute in some way to issues with medicines, e.g., inexperience, stress, personality, attitudes.
7. Medication policies and processes	Policies/directives that impact on the safety of medication usage.
8. Medication safety culture	Organizational values, beliefs, and practices surrounding the management of medication safety and learning from error.
9. Patient- and carer/relative-related factors a. Patient knowledge b. Patient responsibility c. Patient involvement d. Physical and cognitive	Features of the patient that make involvement in safe use of medicines more difficult and therefore more prone to error (e.g., abnormal physiology, language difficulties, personality). Patient condition affects safety or impact of safety issues.
10. Role and responsibilities	Existence of clear lines of responsibility clarifying accountability of staff members and delineating the job role when dealing with medicines (complaints and lack of clarity around lines of responsibility).
11. Supplies of medication and appliances	Issues surrounding obtaining timely supplies of medicines or appliances.
12. Workload of health care professionals	Perceived level of activity and pressures on time during working hours.

**Table 2:** Themes, sub-themes, and illustrative quotes.

Theme	Sub-Theme	Illustrative Quote (Abbreviated)	Frequency ^1^
**Access to services**	Long waiting times	“I had hellish migraines every month for years… Ten years later, they realized…”	★★★★★
	Brief consultation times	“…physicians have only ‘five minutes”	★★★★
	Rural access disparities	“I went to 3 gynecological emergency services; from one I was sent away and humiliated, at another they said it’s not urgent and refused me, at a third they said they wouldn’t prescribe it.”	★★★
	Inadequate pre-prescription testing	“You don’t need a damn blood test before they prescribe you birth control pills.”	★★★
	Telemedicine/follow-up access barriers	“It is not possible to get a personal consultation—only a telephone consultation. Based on a phone call, they prescribed me medication.”	★★
**Communication**	Incomplete medication information	“Dear Doctor… how long can Normix tablets be taken?”	★★★★★
	Conflicting information from multiple sources	“…the patient information leaflet specifically states that this medication is not recommended in such cases!”	★★★★
	Disrespectful communication	“The most humiliating experience of my life was related to a female gynecologist.”	★★★★
	Absence of follow-up communication channels	“My doctor is not reachable. No other doctor deals with it—I am waiting for your reply.”	★★★
**Less frequently mentioned**	Medication shortages	“Unfortunately, since it is not available here in Hungary, not everyone can afford it.”	★★
	Healthcare professional factors (burnout, workload)	“I did not go for a smear test for 10 years because of this—it was a bad, traumatizing experience.”	★★
	Roles & responsibilities (physician vs. pharmacist)	“The doctor prescribed it for me without consulting the pharmacist, and it turned out the two medications should not be taken together.”	★★

^1^ = Relative frequency is shown on a five-level ordinal scale (one to five marks) reflecting the coding density of each sub-theme within the corpus; it denotes qualitative prevalence rather than exact counts.

**Table 3:** Summary of study limitations, their potential impact, and directions for future research.

Limitation	Potential Impact on Findings	Mitigation/Future Direction
Digital-divide/selection bias	Public online data underrepresent older, lower-digital-literacy and lower-SES patients—groups at higher risk of medication harm	Triangulation with interviews, surveys, or pharmacy- and primary-care-based studies
Negativity bias of online discourse	Forums favour negative experiences, potentially overrepresenting system failures	Designs that also capture positive or routine encounters
Lack of clinical validation	Reported incidents reflect patients’ subjective accounts and cannot be confirmed as actual errors	Linkage with clinical records where ethically feasible
Absence of demographic data	No age, sex, or socio-economic information, precluding subgroup analysis	Demographically informed sampling
Pandemic data period (2020–2023)	COVID-19 disruptions likely amplified reported access barriers	Re-examination of post-pandemic discourse
Focus on oral medications	Excludes injectables, inhalers, and biologics with different risk profiles	Separate investigation of non-oral routes
No pharmacist-specific sub-analysis	“Pharmacist-blindness” rests on the qualitative role-comment pattern, not a targeted frequency analysis	Dedicated pharmacy-term keyword search and stratified coding

## Data Availability

The processed data presented in this study are available on request from the corresponding author due to ethical reasons.

## Abbreviations

The following abbreviations are used in this manuscript:

ATC	Anatomical Therapeutic Chemical (ATC) Classification System
COM-B (model)	Capability, Opportunity, Motivation, and Behavior (model)
EESZT	National eHealth Infrastructure
GP	General Practitioner
HCP	Healthcare Professional (or Provider/Personnel)
NEAK	National Health Insurance Fund
SZEBB Programme	Necessary, Effective, and Safe Patient Cooperation in Pharmacies
WHO	World Health Organization
